# Psychological rapid response to population movements in democratic republic of congo (DRC)

**DOI:** 10.1192/j.eurpsy.2021.863

**Published:** 2021-08-13

**Authors:** E. Dozio, C. Bizouerne

**Affiliations:** 1 Mental Health And Care Practices, Action Contre la Faim, Paris, France; 2 Mental Health And Care Practices, Gender And Protection, Action contre la Faim, Paris, France

**Keywords:** Democratic Republic of Congo (DRC), short psychological intervention, PTSD, humanitarian

## Abstract

**Introduction:**

In DRC, the North Kivu province has been plagued by recurring humanitarian crises for nearly two decades, with multiple displacements of populations triggered low intensity armed inter-communal conflicts spread throughout nearly all territories. 818,605 people (displaced, returnees and indigenous) affected by these movements are in alarming psychosocial vulnerability.

**Objectives:**

In 2019, the NGO Action contre la Faim started a psychological intervention with the objective to contribute to reducing the vulnerability of conflict-affected populations.

**Methods:**

Participants have been identified through psychoeducation sessions in the community in which people recognizing corresponding symptoms in themselves were evaluated through a short one-on-one interview. Persons identified as particularly in distress, including those who have experienced gender-based violence, have been involved in a short group therapeutic intervention. Two different options have been proposed in order to evaluate the most effective for the specific context: six sessions with a weekly or bi-weekly frequency.

**Results:**

767 people participated in the psychosocial intervention, 457 with weekly frequency and 310 bi-weekly. The measures of post traumatic symptoms (PCL-5), anxiety and depression (HAD) and resilience (CD-RISC) show that the two approaches have the same positive effects. This is very important in volatile contexts with difficulties of access to the population due to security problems.

**Conclusions:**

The fact that even a short intervention focused on a bi-weekly frequency, can reduce the distress and increase the psychological resilience of populations living in contexts of conflict, gives us the possibility of intervening in areas with limited access, while guaranteeing therapeutic efficacy.

